# Ferumoxytol-β-glucan Inhibits Melanoma Growth via Interacting with Dectin-1 to Polarize Macrophages into M1 Phenotype

**DOI:** 10.7150/ijms.61525

**Published:** 2021-06-26

**Authors:** Xinghan Liu, Yujun Xu, Yi Li, Yuchen Pan, Shuli Zhao, Yayi Hou

**Affiliations:** 1 The State Key Laboratory of Pharmaceutical Biotechnology, Division of Immunology, Medical School, Nanjing University, Nanjing 210093, China.; 2 General Clinical Research Center, Nanjing First Hospital, Nanjing Medical University, Nanjing 210006, China.; 3 Jiangsu Key Laboratory of Molecular Medicine, Nanjing University, Nanjing 210093, China.

**Keywords:** FMT, β-glucan, macrophage, Dectin-1, melanoma

## Abstract

**Background:** Regulating the polarization of macrophages to antitumor M1 macrophages is a promising strategy for overcoming the immunosuppression of the tumor microenvironment for cancer therapy. Ferumoxytol (FMT) can not only serve as a drug deliver agent but also exerts anti-tumor activity. β-glucan has immuno-modulating properties to prevent tumor growth. Thus, a nanocomposite of FMT surface-coated with β-glucan (FMT-β-glucan) was prepared to explore its effect on tumor suppression.

**Methods:** Male B16F10 melanoma mouse model was established to explore the antitumor effect of FMT-β-glucan. The viability and apoptotic rates of B16F10 cells were detected by cell counting kit-8 and Annexin-V/PI experiments. The levels of M1 markers were quantified by quantitative reverse transcription-polymerase chain reaction and enzyme linked immunosorbent assay. Phagocytic activity and intracellular reactive oxygen species (ROS) in macrophages were evaluated by the neutral red uptake assay and flow cytometry, respectively. Small interfering RNA (siRNA) transfection was applied to knock down the Dectin-1 gene in RAW 264.7 cells.

**Results:** FMT-β-glucan suppressed tumor growth to a greater extent and induced higher infiltration of M1 macrophages than the combination of FMT and β-glucan (FMT+β-glucan) *in vivo*. *In vitro*, supernatant from FMT-β-glucan-treated RAW 264.7 cells led to lower cell viability and induced more apoptosis of B16F10 cells than that from the FMT+β-glucan group. Moreover, FMT-β-glucan boosted the expression of M1 type markers, and increased phagocytic activity and ROS in RAW 264.7 cells. Further research indicated that FMT-β-glucan treatment promoted the level of Dectin-1 on the surface of RAW 264.7 cells and that knockdown of Dectin-1 abrogated the phosphorylation levels of several components in MAPK and NF-κB signaling.

**Conclusion:** The nanocomposite FMT-β-glucan suppressed melanoma growth by inducing the M1 macrophage-activated tumor microenvironment.

## Introduction

The latest estimated data on melanoma showed nearly 100,350 new cases and 6850 deaths occurring in the United States in 2020, as the fifth most common cancer in males and females 1. The incidence of melanoma has exhibited a continuous increase over the past 40 years, with slight improvement in long-term survival 2. However, the survival rate of patients with melanoma remains low. After surgical resection, approximately 30% of the patients develop metastasis in various organs. Patients carrying metastatic melanoma have a bad prognosis, with a five-year survival rate between 5-19% 3. Compared with other tumors, melanoma is considered an immunogenic malignancy because of the large number of tumor-infiltrating lymphocytes 4. Based on these findings, immunotherapies, such as immune checkpoint inhibitors and cytokines, have revolutionized the strategies for melanoma treatment 5. Several drugs, such as ipilimumab, pembrolizumab and bempegaldesleukin have been approved for use in the first-line setting of advanced melanoma 6, 7. Besides low survival rates for a substantial number of patients, problems such as low clinical response, drug resistance, and high drug prices hinder the implementation of immunotherapies 8, 9. Therefore, seeking and developing more effective and practical approaches for melanoma treatment is urgent needed.

Ferumoxytol (FMT), an FDA-approved iron oxide nanoparticle, is widely used to treat iron deficiency anemia 10, 11. In a previous phase III clinical study, the intravenous iron replacement therapy with FMT was well tolerated in the patients and achieved the expected therapeutic effect without any gastrointestinal symptoms or hypotension 12. Given its strong safety and unique characterization to label macrophages, FMT has been used to identify inflammation status and monitor therapy-mediated changes in inflammatory diseases in magnetic resonance imaging 13, 14. Moreover, the potential value of FMT in tumor therapies has recently gained great interest from researchers. Although the clinical dose of FMT had no direct tumoricidal effect, coinjection of cancer cells and FMT markedly suppressed tumor development and metastasis in the liver and lungs by increasing M1 polarization of macrophages in a murine tumor model 15. In addition, FMT combined with other immunologic agents (Toll-like receptor (TLR) 3 agonist (poly(I:C)) or TLR 9 agonist (CpG)) exerted stronger antitumor activity than FMT used alone 16, 17. Thus, we should explore the synergy of FMT and other immune activators in order to develop more effective treatment strategies for melanoma.

β-glucan is a type of natural polysaccharide that can be extracted from a variety of plants such as oats, barley, and seaweed and is also the component of the cell walls of some pathogenic bacteria (Pneumocystis carinii, Cryptococcus, Aspergillus fumigatus, and Candia albicans) and fungi (Saccaromyces cerevisiae). Although different kinds of β-glucan are all glucan polymers connected by β-(1,3) and (1,4) or (1,6) glycosidic chains, their diversities of length and branch structures determine the difference of their extraction method and biological activity 18. Studies have shown that β-glucan with large, intermediate or low molecular weight, or yeast-derived β-glucan has biological activity *in vivo*, however short β-glucan (molecular weight less than 5000-10000) is normally inactive 19, 20. β-glucan is considered an important anticancer agent because of its regulation of immune cells, anti-angiogenesis, enhanced sensitivity to radiotherapy and chemotherapy, and direct cytotoxicity to tumor cells 21, 22. The *in vitro* studies found yeast-derived β-glucan could be conjunct to the lectin sites of complement receptor 3 (CR3) of neutrophils, macrophages, and natural killer (NK) cells and mediate the cytotoxic effects of these cells against tumors opsonized with iC3b 23, 24. Vaclav Vetvicka et al. find oral administration of Saccharomyces cerevisiae-derived β-glucan strongly reduced the tumor weight and lung metastasis, and prolonged the overall survival of mice carrying melanoma *in vivo* via activating NK cells, and enhanced the efficacy of regular chemotherapeutic drugs 25. In addition, β-glucan can alter the immune-suppressive tumor microenvironment. On the one hand, β-glucan can switch monocytic myeloid-derived suppressor cells (M-MDSCs) to antigen presenting cells, resulting in the activation of CD4^+^ and CD8^+^ T cells, and the expression of proinflammatory cytokines (interferon (IFN)-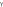, tumor necrosis factor (TNF)-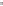, and perforins), and further damaging tumor cells 26. On the other hand, oat- or yeast-derived β-glucan induces macrophages polarization from the suppressive M2 status to the tumoricidal M1 phenotype by Dectin-1 or TLR2, then activates Th1 type T cells, and finally suppresses the growth of melanoma and lung cancer 27, 28. Additionally, pretreatment of mice with β-glucan can establish trained immunity, thereby rewiring the transcriptomics and epigenetics of granulopoiesis and reprogramming neutrophils toward anti-tumor phenotype, and ultimately diminishing tumor growth 29. In addition to the above functions, β-glucan can also directly induce apoptosis of some cells and released reactive oxygen species (ROS) will kill tumor cells irrespective of the immune system. Recently, it was reported that water-soluble β-D-glucan directly reduced hepatocellular carcinoma (HCC) cell growth *in vitro* and* in vivo*, and the effect was significantly linked with autophagy blockage by β-D-glucan and thereby mitochondria dysfunction and excess ROS, which promoted the apoptosis of HCC cells under the lack of nutrition 30.

Consequently, whether the combination of FMT and β-glucan (FMT+β-glucan) or the nanocomposite of FMT surface-coated with β-glucan (FMT-β-glucan) has a synergistic effect on the elimination of tumors and the regulation of macrophages prompted us to conduct subsequent research. In this study, we identified that the nanocomposite FMT-β-glucan effectively inhibited melanoma growth by inducing the polarization of macrophages into proinflammatory M1 macrophages and displayed greater therapeutic benefit than the treatment of FMT combined with β-glucan. Furthermore, by binding to the Dectin-1 receptor on the surface of macrophages, FMT-β-glucan polarized macrophages into the M1 type partly through activating the mitogen-activated protein kinase (MAPK) and spleen associated tyrosine kinase (Syk)/nuclear factor kappa-B (NF-κB) signaling pathways.

## Methods

### Materials

β-glucan used in this study was purchased from Sigma (G-5011), which was hereinafter referred to as β-glucan for short. β-glucan with a β-(1,3)-glucan linear structure and a small number of β-(1,6)-glucan branches was extracted from Saccaromyces cerevisiae and had been used in other researchers' studies 31, 32. The preparation method and characterization of β-glucan has been described in the previous studies 22, 33, and the purity of β-glucan is greater than 98%.

### Synthesis of FMT surface-coated with β-glucan (FMT-β-glucan)

FMT was prepared by pathways described in previous reports 34. To prepare FMT-β-glucan, FMT, β-glucan (Merck KGaA, Darmstadt, Germany), and 1-ethyl-3-(3-dimethylaminopropyl) carbodiimide hydrochloride (EDC) (J&K Scientific Ltd, Shanghai, China) were added to appropriate deionized water and stirred overnight at room temperature. Excess β-glucan was removed by dialyzing with a dialysis bag (300 kDa MWCO) for 48 h. Finally, the mixture was lyophilized into powder and used for subsequent experiments.

### Characterization of FMT-β-glucan

Infrared absorption spectroscopy was used to confirm the successful binding of β-glucan to the FMT surface. The content of Fe in FMT was measured by ICP-OES (Optima 8000, PerkinElmer, USA). The particle sizes and zeta potentials of FMT and FMT-β-glucan were assessed using the dynamic light scattering technique (Malvern, UK). Transmission electron microscopy (TEM; JEM-1200EX, JEOL, Japan) was applied to observe the morphology of FMT and FMT-β-glucan. To evaluate the stability in diverse pH conditions and serum, the FMT-β-glucan was cultured in solutions with several pH values of 5.5, 6.8 and 7.4 or DMEM medium (Gibco, Carlsbad, CA) containing 10% fetal bovine serum (FBS; Gibco, Carlsbad, CA) at 37 °C and the particle sizes were determined by dynamic light scattering technique.

### Rhodamine B (RhB)-labeled FMT-β-glucan

Briefly, RhB (0.5 mg; Sigma, USA), FMT-β-glucan (20 mg), and dimethyl sulfoxide (DMSO; J&K Scientific Ltd) (5 mL) were mixed and stirred in an Erlenmeyer flask overnight at ambient temperature. Then the mixture was dialyzed with distilled water by a dialysis bag (3.5 kDa MWCO) for 48 h and lyophilized into powder.

### *In vitro* drug release

We evaluated the release of β-glucan from FMT-β-glucan under various pH values (5.5, 6.8 and 7.4) *in vitro*. First, we prepared the RhB-labeled β-glucan using the above instruments. Then a dialysis bag (300 kDa MWCO) with 5 mL of FMT-β-glucan solution was placed in a PBS solution and shaken in the dark using a 37 °C shaker. Finally, the dialysate was removed at selected time points, and the release profile was assessed by fluorescence.

### Mouse tumor model

All of the animal experiment protocols had reached the approval of the Institutional Animal Care and Use Committee of Nanjing First Hospital, Nanjing Medical University (DW20200204) and were conformed to the guides issued by the National Institutes of Health. Male C57BL/6 mice (5-6 weeks old) were bought from the Nanjing Model Animal Institute (Nanjing, China). A subcutaneous tumor mouse model was constructed by subcutaneously injected with 100 µL sterile PBS containing 5 × 10^5^ B16F10 cells in the right flank of mice. Mice were randomly distributed into five groups when tumors reaching a volume of about 100 mm^3^, and intraperitoneally administrated with PBS, FMT (400 µg), β-glucan (80 µg), FMT+β-glucan (400 µg FMT + 80 µg β-glucan) and FMT-β-glucan, containing FMT 400 µg and β-glucan 80 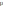
g, every day. Tumor growth was observed via magnetic resonance imaging (MRI; Biospec 7T/20USR GmbH Bruker, Germany). The parameters were set as follows: TR = 2500 ms, TE = 33 ms, FA = 180 deg, and field of view (FOV) = 3.5 cm. Body weight and tumor size were measured and recorded every other day by scales and Vernier calipers. The following formula was applied to calculate the tumor volume: volume (V) = length (L) × width (W)^2^ × 1/2.

### Analysis of tumor-infiltrating immunocytes

Scissors and tweezers were used to carefully peel off the tumor tissues. After weighing, tumor tissues were harvested and digested with RPMI 1640 medium containing 5% FBS, 2 mg/mL collagenase IV (Sigma, USA) and 5 U/mL DNase I (Sigma, USA) for 20 minutes by a 37 °C shaker. Then the cell suspension was neutralized with RPMI 1640 medium containing 5% FBS and filtered through 70-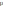
m nylon mesh into a centrifuge tube to obtain single cell suspension. Bone marrow cells were obtained as described above. Spleens were milled and filtered with PBS into centrifuge tubes through a 200-mesh sieve. Then, the cells were lysed with red blood cell lysate, filtered through gauze, and resuspended in PBS. For immunocyte detection, the following anti-mouse antibodies were used: CD45-PE (103106, BioLegend), CD45-APC (17-0451-83, eBioscience), CD11b-APC (101212, BioLegend), CD11b-PerCP (45-0112-82, eBioscience), Gr-1-FITC (108406, BioLegend), F4/80-FITC (11-4801-82, eBioscience), NOS2-PE (12-5920-82, eBioscience), CD206-PE (12-2061-82, eBioscience), and CD206-APC (17-2061-82, eBioscience). A FACSCalibur flow cytometer (Becton Dickinson, Franklin Lakes, NJ) was applied to detect the stained cells and the FlowJo software (TreeStar, Ashland, OR) was utilized to analyze the data.

### Immunohistochemistry (IHC)

Harvested tumor tissues were kept in the 4% paraformaldehyde solution (Servicebio, Wuhan, China) and then embedded in paraffin. Antibodies against Ki67, F4/80, CD86 and CD206 antibody were used as primary antibodies. All sections were imaged via a light microscope (ECLIPSE Ti-U, Nikon, Japan), and the ImageJ software (National Institutes of Health, USA) was used for the analysis of images.

### Enzyme-linked immunosorbent assay (ELISA)

The supernatants of RAW 264.7 cells or blood from mice were collected to determine TNF-α or interleukin (IL)-6 expression by a commercial mouse TNF-α ELISA kit (4A BiotechCo. Ltd, Beijing, China) and a mouse IL-6 ELISA kit (Neobioscience, Shanghai, China), following the instructions in the manual.

### Cell culture and chemicals

RAW 264.7 cells, B16F10 melanoma cells and ID8 mouse ovarian cancer cells were obtained from Fudan Cell Bank and kept in Dulbecco's modified Eagle's medium (DMEM) or RPMI 1640 medium containing 10% FBS and 1% v/v penicillin and streptomycin (Gibco, Carlsbad, CA) at 37 °C in an incubator with 5% CO_2_.

### Cell viability

The cell viability was detected by a Cell Counting Kit-8 (CCK-8) following the instructions in the manual 35. RAW 264.7 cells, B16F10 and ID8 cells were cultured in 96-well plates at appropriate densities (for the detection of cell viability in a coculture system, B16F10/ID8 and RAW 264.7 cells were mixed in a 1:2 ratio) and treated with FMT, β-glucan, or a combination of both for 48 h.

### Cell proliferation analysis

First, B16F10 and ID8 cells, and RAW 264.7 cells were prelabeled with the dye CFSE (DOJINDO, Shanghai, China) and eFluor 670 (eBioscience) following the manufacturer's instructions, respectively. Then tumor cells and RAW 264.7 cells were directly or indirectly cocultured at the ratio of 1:2 and treated with 400 µg/mL FMT, 80 µg/mL β-glucan and 400 µg/mL FMT+ 80 µg/mL β-glucan for 48 h. For cell proliferation analysis, the cells were analyzed by FCM. Mod Fit LT 3.0 software was applied to calculate the proliferation index (PI) of tumor cells. The growth inhibition rate of tumor cells was measured according to the following equation: cell growth inhibition rate = (1-(PI (treatment group)/ PI (control group) ) × 100%.

### Cell apoptosis analysis

B16F10 cells were treated with FMT+β-glucan and FMT-β-glucan for 48 h. Then the cells were harvested and incubated with staining solution that comprised of 195 µL binding buffer, 5 µL Annexin V, and 2.5 µL PI for 15 minutes at ambient temperature without light. Finally, the cells were resuspended in binding buffer solution and subjected to flow cytometry (FCM) analysis.

### Quantitative reverse transcription-polymerase chain reaction (qRT-PCR)

TRIzol reagent (Invitrogen, Grand Island, NY, USA) was employed to obtain total RNA from treated cells. Then, 1 µg of the collected RNA was reverse-transcribed to cDNA following the protocol of a HiScript II Q RT SynthesisMix Kit (Vazyme, Nanjing, China), and cDNA was subjected to PCR quantification by SYBR Green SuperMix reagent (Bio-Rad, California, USA). Finally, the levels of genes of interest were quantified by the 2^-ΔΔCt^ method based on its relative expression to GAPDH. Detail sequences of the primers are available in Table 1.

### Neutral red uptake assay

Treated cells cultured in a 96-well plate were stimulated for 1 h with 200 µL/well physiological saline solution containing 0.1% neutral red and washed with mild phosphate buffered solution (PBS) for 3 times. Then 200 µL cell lysate prepared in an equal volume of absolute ethanol and acetic acid was added into each well, and the cells were set aside for at least 3 h at room temperature. Finally, the absorbance values of each well were examined at 540 nm.

### Detection of reactive oxygen species (ROS)

After stimulation for 12 h, cell culture supernatants were discarded and an appropriate volume of serum-free medium with a final concentration of 10 µM of 2,7-dichlorodihydrofluorescein diacetate (DCFH-DA) (Beyotime, Shanghai, China) was added. Then the cells were incubated for 20 minutes at 37 °C without light and washed in serum-free medium for 3 times to fully remove the left DCFH-DA. Finally, the cells were subjected to FCM analysis.

### Western blotting (WB) analysis

RIPA lysis buffer (Beyotime, Shanghai, China) was utilized to obtain the total proteins. After being quantified by a BCA protein assay kit (Thermo Fisher Scientific, Waltham, MA), equal proteins were loaded and electrophoresed on a polyacrylamide gel, and wet-transferred to PVDF membranes (Millipore, Bedford, MA). Then the membranes were kept in 5% bovine serum albumin (BSA) in Tris-buffered saline with 0.1% Tween 20 (TBST) at indoor temperature for 1.5 h and kept in primary antibody solution with a 1:1000 dilution ratio at 4 °C for 12 h. After being treated with secondary antibody solution with a 1:5000 dilution ratio for 1 h at ambient temperature, the membranes were observed with ECL Plus WB detection reagents (Millipore).

The primary antibodies including mouse anti-phosphorylated (p)-p38, mouse anti-p38, rabbit anti-p-extracellular regulated MAP kinase (p-ERK), rabbit anti-ERK, rabbit anti-p-c-Jun NH2-terminal (p-JNK), rabbit anti-JNK, rabbit anti-β-actin, rabbit anti-p-Syk, rabbit anti-Syk, rabbit anti-p- protein kinase C delta (PKC δ), rabbit anti-PKC δ, rabbit anti-p-IKK α/β, rabbit anti-IKK α, rabbit anti-p-P65, and rabbit anti-P65 were bought from Cell Signaling Technology (Danvers, Massachusetts, USA). The rabbit anti-Histone 3 antibody was bought from Abcam (Cambridge, Massachusetts, USA).

### Immunofluorescence staining

The transportation of P65 to the nucleus was detected by immunofluorescence staining to confirm whether NF-κB pathway was activated, according to the protocol of a nuclear translocation assay kit (Beyotime, Shanghai, China), as described previously 36.

### Uptake of FMT-β-glucan by macrophages *in vitro*

RAW 264.7 cells were treated with RhB-labeled FMT-β-glucan for 6 h or 24 h and the uptake of FMT-β-glucan by RAW 264.7 cells was observed by FCM or FV3000 laser scanning confocal microscopy (LSCM; lympus, Japan) after staining the nuclei with 4,6-diamino-2-phenyl-indole (DAPI) for 5 minutes.

### Small interfering RNA (siRNA) transfection

siRNA oligonucleotides for Dectin-1 and a noncoding (NC) siRNA with no biological effects were obtained from GenePharma (Shanghai, China). siRNA transfection was performed according to the product instructions at a concentration of 50 nM. After transfection for 24 h and 48 h, qRT-PCR and WB assays were used to detect the knockdown efficiency of Dectin-1.

### Statistical analysis

Prism 6 (GraphPad Software, Inc., San Diego, CA) was applied to perform the statistical analysis by Student's t-test or one-way ANOVA. Significant differences were marked as ^*^p < 0.05 and^ **^p < 0.01.

## Results

### FMT+β-glucan has an inhibitory effect on tumor growth by regulating macrophages *in vivo*

**Figure 1:**
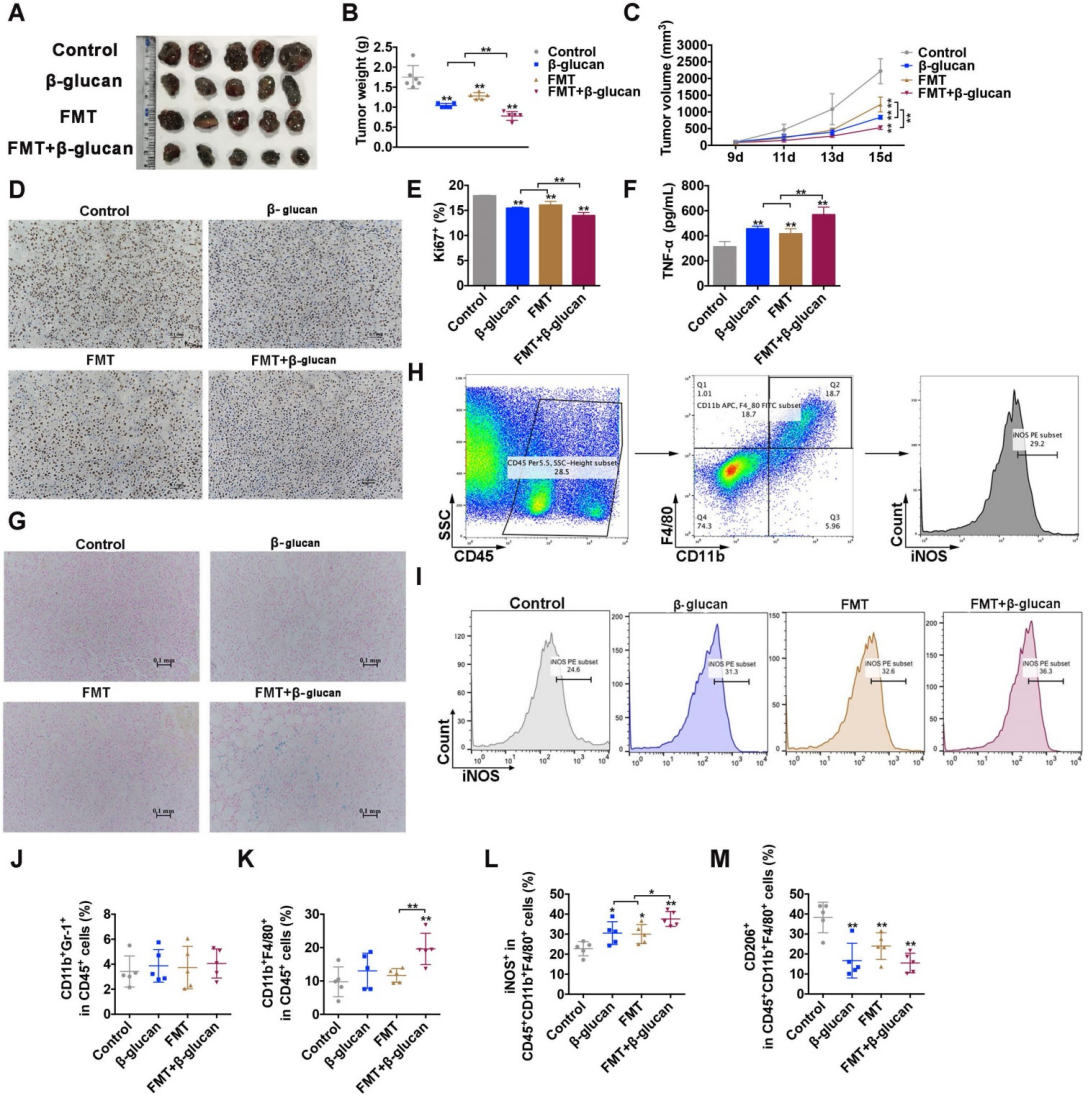
**FMT+β-glucan suppressed tumor growth and regulated macrophages *in vivo***. (A) Photograph of dissected tumor tissues from mice with the indicated treatments. (B) Tumor weight of dissected tumor tissues from mice with the indicated treatments. (C) Tumor volume of mice during the indicated treatment. (D) Histopathologic photograph of Ki67 staining of tumor tissues from mice with the indicated treatments. (E) Quantitative data of D. (F) The expression of TNF-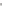 in the blood serum of mice with the indicated treatments. (G) Images of Prussian blue staining of dissected tumor tissues from mice with the indicated treatments. (H) Schematic diagram of the gating principle in the FCM. (I) FCM photographs of iNOS expression in the tumor tissues with the indicated treatment. (J-M) Quantitative data of FCM analysis of MDSCs (CD45^+^CD11b^+^Gr-1^+^; J), macrophages (CD45^+^CD11b^+^F4/80^+^; K), M1 (CD45^+^CD11b^+^F4/80^+^iNOS^+^; L) and M2 (CD45^+^CD11b^+^F4/80^+^CD206^+^; M) macrophages in the tumor tissues with the indicated treatments. * p < 0.05, ** p < 0.01.

First, we assessed the effect of FMT, β-glucan and their combination (FMT+β-glucan) on tumor growth *in vivo* of male melanoma bearing C57BL/6 mice. As depicted in Figure 1A-C, intraperitoneal injection of FMT or β-glucan remarkably delayed tumor progression via reduced tumor weight and tumor volume, and coadministration of FMT plus β-glucan significantly improved the therapeutic effect. Immunohistochemical analysis of Ki67 in tumor tissues also found that FMT or β-glucan treatment reduced tumor proliferation, and co-administration notably amplified the effect (Figure 1D and 1E). Furthermore, the Prussian blue staining showed that FMT+β-glucan could accumulate in the tumor tissues (Figure 1G).

Previous studies reported that FMT or β-glucan might induce macrophage polarization *in vitro* and *in vitro*, thus the distribution and subgroups of macrophages in tumor tissues, bone marrow cells and splenocytes were examined. FCM results indicated that treatment with FMT+β-glucan did not alter the infiltration of MDSCs (CD45^+^CD11b^+^Gr-1^+^) compared to that in the control group and solo treatment, but increased the ratio of total macrophages (CD11b^+^F4/80^+^ in CD45^+^ cells) and M1-type macrophages in tumor tissues (Figure 1H-L). M2-like macrophages were significantly decreased in any treatment groups (Figure 1M). Meanwhile, a significant difference was also found in bone marrow cells and splenocytes by administration of FMT+β-glucan. The percentage of total macrophages in bone marrow cells and the ratio of total and M1 phenotype macrophages in splenocytes increased markedly, while the proportion of M2-type macrophages sharply decreased in both (Figure S1). In addition, systemic administration of FMT+β-glucan increased the expression of TNF-α in the blood serum of mice (Figure 1F). Therefore, these results together demonstrated that FMT+β-glucan resulted in a macrophage-activated tumor microenvironment, which possibly contributed to durable melanoma regression.

### Preparation and characteristics of FMT-β-glucan

**Figure 2:**
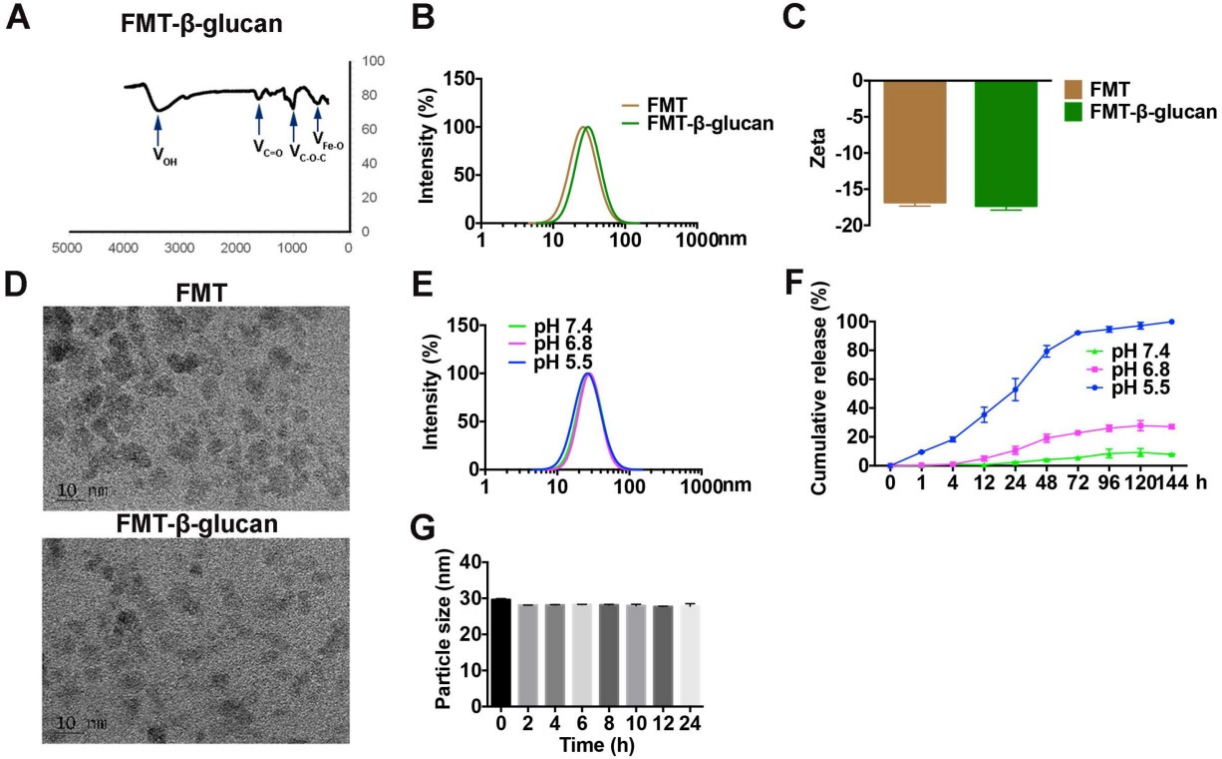
** Characteristics of FMT-β-glucan.** (A) The successful binding of β-glucan to the FMT surface was confirmed by infrared absorption spectroscopy. (B) Particle size of FMT and FMT-β-glucan. (C) Zeta potential of FMT and FMT-β-glucan. (D) Images of FMT and FMT-β-glucan were obtained by TEM. (E) Changes in the particle size of FMT-β-glucan at different pH conditions. (F) Release profiles of β-glucan from FMT-β-glucan under different pH conditions. (G) Particle sizes of FMT-β-glucan after incubation for different durations in DMEM supplemented with 10% FBS at 37 °C.

For simultaneous delivery of FMT and β-glucan, an engineered nanocomposite (FMT-β-glucan) was prepared. As observed in Figure 2A, the surface of FMT was successfully coated with β-glucan. The particle size and zeta potential of FMT-β-glucan, detected by dynamic light scattering, can be found in Figure 2B and 2C. FMT and FMT-β-glucan nanoparticles were homogeneously dispersed as revealed by TEM (Figure 2D). We then assessed the stability of FMT-β-glucan at various pH conditions by measuring particle sizes. The particle size of FMT-β-glucan increased slightly when the pH value of the solution decreased from pH 7.4 to 5.5, suggesting that the nanocomposite might release β-glucan and FMT under the acidic conditions (Figure 2E). We further assessed the release profile of β-glucan from FMT-β-glucan *in vitro* and found that β-glucan was released faster in the pH 6.8 medium than in pH 7.4 medium (Figure 2F). Under the pH 5.5 conditions, approximately 50% of β-glucan was released from the nanocomposite after 24 h and 90% was released after 72 h (Figure 2F). In addition, the particle sizes of FMT-β-glucan had no obvious change during 24 h incubation with 10% FBS solution, which indicated that MT-β-glucan was suitable for systemic injection (Figure 2G).

### FMT-β-glucan displays a more notable inhibitory effect on tumor growth than by FMT+β-glucan by regulating macrophages *in vivo*

**Figure 3:**
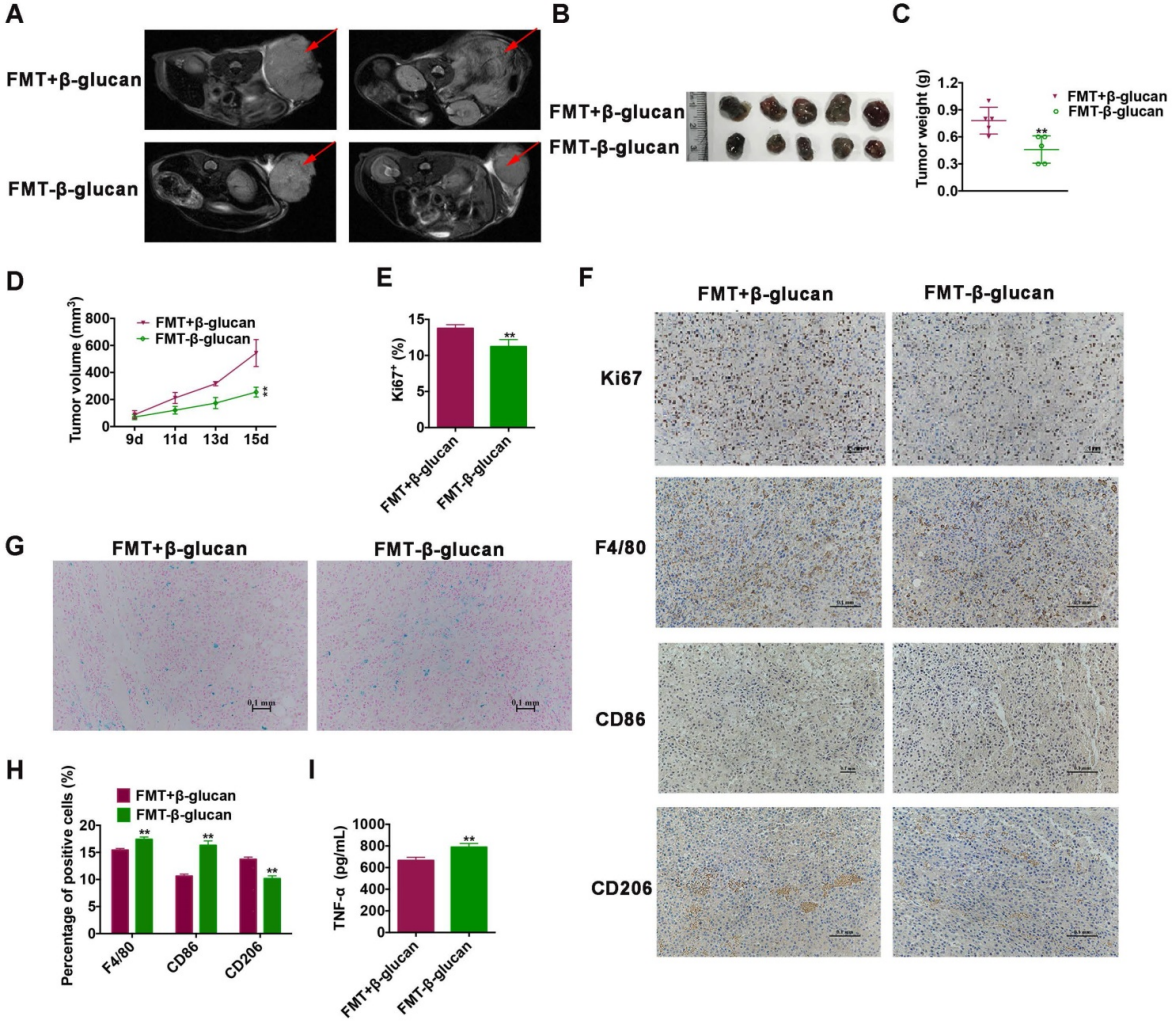
** FMT-β-glucan suppressed tumor growth to a greater extent than FMT+β-glucan by polarizing macrophages *in vivo*.** (A) MRI images of mice with FMT+β-glucan or FMT-β-glucan treatment. (B) Photograph of dissected tumor tissues from mice with the indicated treatments. (C) Tumor weight of dissected tumor tissues from mice with the indicated treatments. (D) Tumor volume of mice during the indicated treatments. (E) Quantitative data of Ki67 staining of tumor tissues from mice with the indicated treatments. (F) Histopathologic photographs of Ki67, F4/80, CD86 and CD206 staining of tumor tissues from mice with the indicated treatments. (G) Images of Prussian blue staining of dissected tumor tissues from mice with the indicated treatments. (H) Quantitative data of F4/80, CD86 and CD206 staining of tumor tissues from mice with the indicated treatments. (I) The expression of TNF-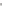 in the blood serum of mice with the indicated treatments. * p < 0.05, ** p < 0.01.

Encouraged by the above results, whether the synthesized nanocomposite (FMT-β-glucan) had the same efficacy of tumor elimination as the combination treatment of FMT+β-glucan was then explored *in vivo*. We surprisingly found that FMT-β-glucan displayed a stronger tumor growth inhibition effect than FMT+β-glucan with reduced tumor weight and tumor volume, as well as decreased Ki67 expression (Figure 3A-3E). In addition, the Prussian blue staining showed that FMT-β-glucan had significantly higher FMT accumulation in tumor tissues than FMT+β-glucan (Figure 3G). Given the polarization of macrophages induced by FMT+β-glucan, the efficacy of FMT-β-glucan was also detected. Consistent with the previous results, FMT-β-glucan treatment also increased the infiltration of macrophages especially M1-type macrophages and decreased M2 counterparts to a greater extent than FMT+β-glucan by IHC experiment (Figure 3F and 3H). ELISA experiment revealed the elevated TNF-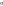 expression in the blood serum of mice administrated with FMT-β-glucan (Figure 3I). Therefore, these results together proved that FMT-β-glucan might have a better antimelanoma effect than the combination of FMT plus β-glucan.

### Effect of FMT-β-glucan on tumor growth inhibition *in vitro*

**Figure 4:**
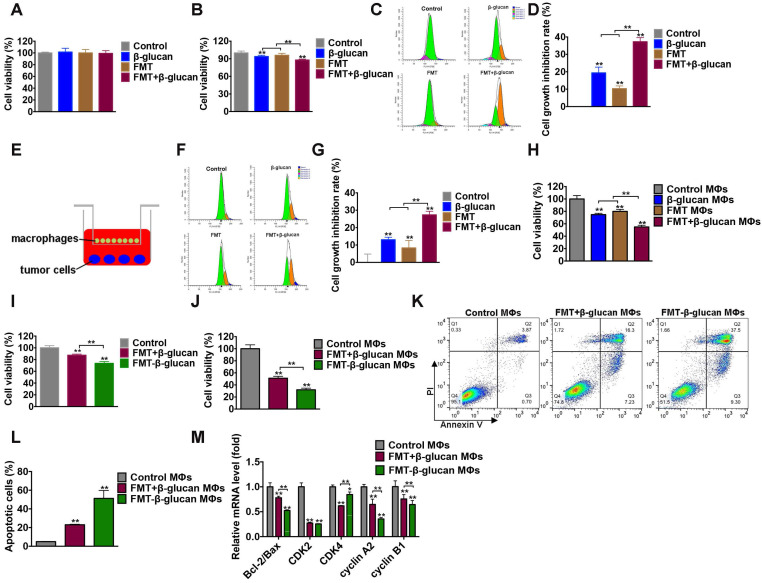
** FMT-β-glucan inhibited tumor growth *in vitro*.** (A) B16F10 cells were treated with FMT, β-glucan, or FMT+β-glucan for 48 h, and the cell viability was examined by CCK-8 assay. (B) In the presence of FMT, β-glucan, or FMT+β-glucan, B16F10 cells were directly cocultured with RAW 264.7 cells for 48 h, and the cell viability was detected by CCK-8 assay. (C) In the presence of FMT, β-glucan, or FMT+β-glucan, B16F10 cells were directly cocultured with RAW 264.7 cells for 48 h, and the cell proliferation of B16F10 cells was detected by FCM. (D) Quantitative data of (C). (E) Schematic diagram of the indirect coculture system. (F) In the presence of FMT, β-glucan, or FMT+β-glucan, B16F10 cells were indirectly cocultured with RAW 264.7 cells for 48 h, and the cell proliferation of B16F10 cells was detected by FCM. (G) Quantitative data of (F). (H) After FMT, β-glucan, or FMT+β-glucan treatment for 24 h, the cell culture supernatants of RAW 264.7 cells were collected and used to treat B16F10 cells for 48 h. The cell viability of B16F10 cells was detected by CCK-8 assay. (I) In the presence of FMT+β-glucan or FMT-β-glucan, the total cell viability of B16F10 cells cocultured with RAW 264.7 cells for 48 h were detected by CCK-8 assay. (J) The viability of B16F10 cells was detected by CCK-8 assay after FMT+β-glucan MΦs or FMT-β-glucan MΦs treatment for 48 h. (K) Apoptotic cells of B16F10 cells were examined by FCM after FMT+β-glucan MΦs or FMT-β-glucan MΦs treatment. (L) Quantitative data of (K). (M) Expression of the cell cycle- and apoptosis-related genes of B16F10 cells after FMT+β-glucan MΦs or FMT-β-glucan MΦs treatment for 24 h was detected by qRT-PCR. * p < 0.05, ** p < 0.01.

Considering the function of FMT-β-glucan in tumor suppression and macrophage regulation* in vivo*, the effect of FMT-β-glucan *in vitro* was determined. First, the synergistic therapeutic role of the combination treatment of FMT and β-glucan was studied. The effects of FMT, β-glucan, and FMT+β-glucan on B16F10 cell viability were determined by incubation for 48 h. No obvious toxicity in any treatment was observed (Figure 4A). The CCK-8 results showed that when B16F10 and RAW 264.7 cells were cocultured, the addition of β-glucan and FMT+β-glucan would reduce cell viability by 6.12% and 12.2%, respectively (Figure 4B). To accurately verify the direct and indirect inhibition rates on B16F10 cell proliferation, B16F10 cells and RAW 264.7 cells were prestained with dyes CFSE and eFluor 670, respectively. As shown in Figure 4C and Figure 4D, in the direct coculture system of B16F10 and RAW 264.7 cells, treatment with FMT or β-glucan only slightly suppressed B16F10 cell growth (19.37% and 10.32%), whereas the additional FMT in the β-glucan-treated system contributed to a more notable growth inhibition of B16F10 cells (37.29%). In the indirect coculture system (Figure 4E), stimulation with FMT or β-glucan or FMT+β-glucan resulted in cell growth inhibition rates of 13.04%, 8.33%, and 27.36%, which were lower than the effect of direct coculture (Figure 4F and 4G). In addition, the supernatant collected from RAW 264.7 cells (MΦs) without or with any stimulation was used to treat B16F10 cells for 48 h. As seen in the Figure 4H, in the presence of FMT+β-glucan MΦs, B16F10 only obtained a cell viability of 54.91%, which was lower than that in FMT MΦs or β-glucan MΦs (74.79% and 80.02%, respectively). Next, the ID8 cells were cocultured with RAW 264.7 cells under conditions similar to those described above, and similar results were found (Figure S2).

Then, the therapeutic potential of FMT-β-glucan on B16F10 cells was examined. FMT+β-glucan was used for comparison. Compared with the FMT+β-glucan group, FMT-β-glucan treatment revealed a more significant reduction in cell viability in the coculture system of B16F10 and RAW 264.7 cells by CCK-8 assay (Figure 4I). The decrease in cell viability could be caused by multiple factors, such as cell cycle arrest and apoptosis. The cell cycle is often dysregulated in tumors and therapies targeting the cell cycle have become an intense subject of research in recent years 37. Apoptosis is a type of programmed cell death, which can be mediated by the caspase-related pathways 38. Thus, FCM, qRT-PCR and WB assays were used to detect changes in the cell cycle and apoptosis. The supernatants of RAW 264.7 cells treated with FMT+β-glucan and FMT-β-glucan were collected and used to incubate B16F10 cells. Supernatants from the FMT-β-glucan group (FMT-β-glucan MΦs) reduced B16 F10 cell viability to a greater extent (31.95%) and induced more apoptosis (51.23%) than those in the FMT+β-glucan group (51.18% and 23.05%, respectively) (Figure 4J-L). As displayed in Figure 4M, decreased cell viability was tightly correlated with the reduction of the ratio of Bcl-2 to Bax and cyclin-dependent kinases (CDK)2, CDK4, cyclin A2, and cyclin B1. In conclusion, although FMT-β-glucan had no direct tumoricidal effect on tumor cells, FMT-β-glucan might suppress tumor growth by activating macrophages.

### FMT-β-glucan promoted M1 polarization, and increased phagocytosis and ROS production in macrophages

**Figure 5:**
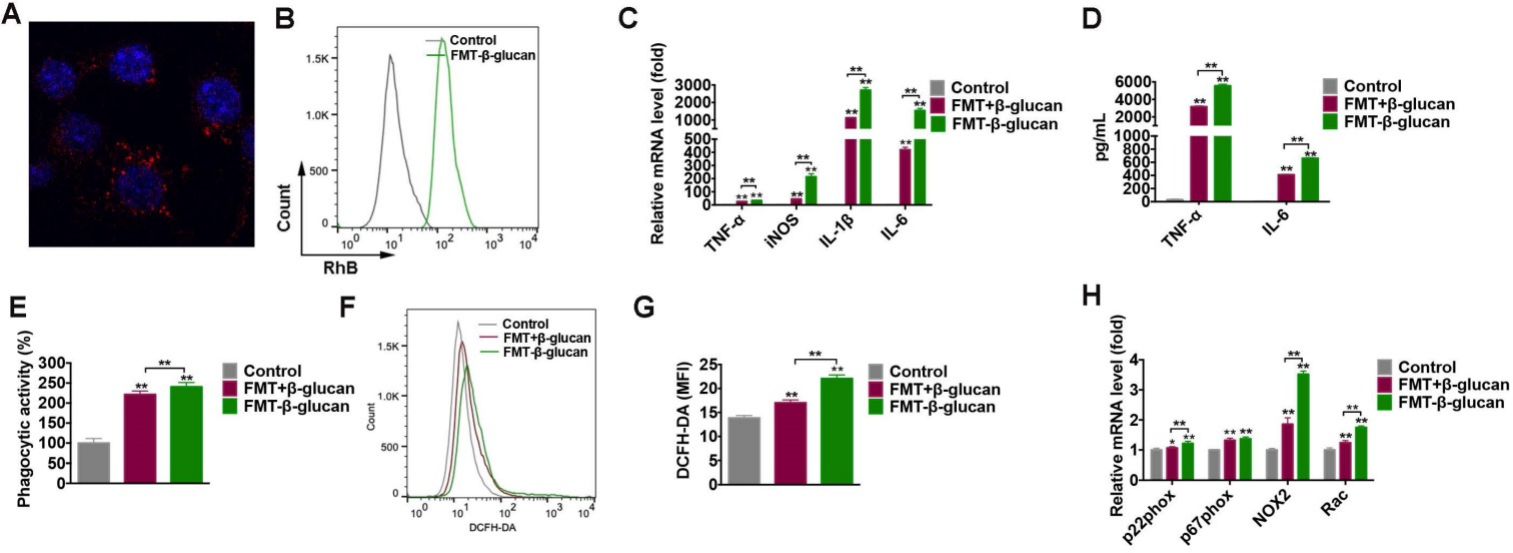
** FMT-β-glucan promoted M1 polarization, and increased phagocytosis and ROS production in macrophages.** (A-B) RAW 264.7 cells were treated with FMT-β-glucan for 24 h or 6 h, and the internalization of FMT-β-glucan by macrophages was detected by FV3000 laser scanning confocal microscopy (A) and FCM (B). (C) After the treatment with FMT+β-glucan or FMT-β-glucan for 6 h, the M1-like gene expression in RAW 264.7 cells was detected by qRT-PCR experiment. (D) RAW 264.7 cells were treated with FMT+β-glucan or FMT-β-glucan for 24 h, and TNF-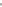 and IL-6 production in the cell culture supernatant was detected by ELISA experiment. (E) Phagocytic activities of RAW 264.7 cells were detected by the neutral red uptake assay after FMT+β-glucan or FMT-β-glucan incubation for 24 h. (F) RAW 264.7 cells were incubated with FMT, β-glucan or FMT+β-glucan for 12 h, and intracellular ROS production was detected by FCM. (G) Quantitative data of (F). (H) RAW 264.7 cells were treated with FMT+β-glucan or FMT-β-glucan for 6 h, and the expression of p22phox, p47phox, NOX2, and Rac was detected by qRT-PCR experiment. *p < 0.05, **p < 0.01.

To verify the internalization of FMT-β-glucan by macrophages, FMT-β-glucan was fluorescently labeled with RhB, and the cellular uptake of FMT-β-glucan was quantified by confocal microscopy and FCM (Figure 5A-5B). Classic M1 type macrophages exert tumoricidal activities partly by secreting cytokines (e.g., tumor necrosis factor (TNF)-α, IL-1β and IL-6), and reactive species of oxygen and nitrogen (e.g., nitric oxide and superoxide) 39. Thus, to investigate the potential role of FMT-β-glucan in polarizing RAW 264.7 cells, the expression of M1-like genes was detected by qRT-PCR after cells were stimulated for 6 h. Compared with controls and FMT+β-glucan, FMT-β-glucan significantly promoted the expression of M1-like genes (TNF-α, iNOS, IL-1β, and IL-6) (Figure 5C) and secretion of TNF-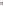 and IL-6 in the supernatants by ELISA assay (Figure 5D).

Previous studies have demonstrated that tumoricidal macrophages are not only characterized by M1 markers, but can also suppress cancer by engulfing tumor cells and producing reactive oxygen species (ROS) 40, 41. The neutral red uptake assay and DCFH-DA were utilized to assess the phagocytic activity and intracellular ROS of RAW 264.7 cells. RAW 264.7 cells treated with FMT-β-glucan had an enhanced phagocytic capacity and increased intracellular ROS, which was slightly higher than that of cells treated with FMT+β-glucan (Figure 5E-5G). Nicotinamide adenine dinucleotide phosphate (NADPH) oxidase, composed of membrane subunits (gp91phox (NOX2) and p22phox), cytoplasmic subunits (p40phox, p47phox, and p67phox) and GTPase binding protein (Rac), is the main enzyme that generates ROS 42. Among these genes, NOX2 was upregulated to the greatest degree following costimulation (Figure 5H). Taken together, FMT-β-glucan polarized macrophages toward a tumoricidal phenotype, with upregulation of M1-like markers, phagocytic capability and intracellular ROS.

### FMT-β-glucan activated macrophages by interacting with Dectin-1 on the surface of macrophages

**Figure 6:**
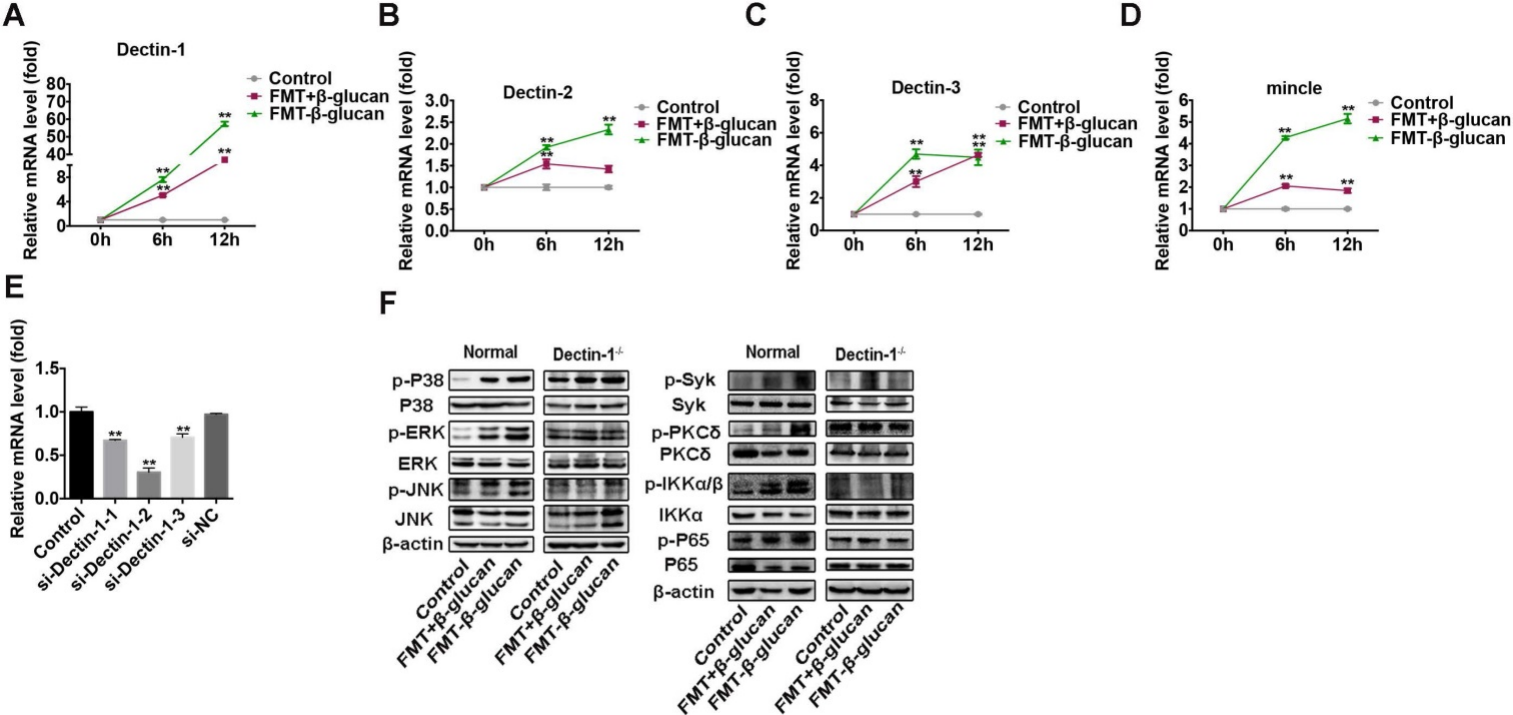
** FMT-β-glucan activated macrophages by interacting with Dectin-1 on the surface of macrophages.** (A-D) RAW 264.7 cells were incubated with FMT+β-glucan or FMT-β-glucan for 6 h, and the expression of Dectin-1 (A), Dectin-2 (B), Dectin-3 (C) and mincle (D) was examined by qRT-PCR assay. (E) The Dectin-1 knockdown efficacy by siRNA transfection was detected by qRT-PCR experiment. (F) RAW 264.7 cells with or without Dectin-1^-/-^ were treated with FMT+β-glucan or FMT-β-glucan for 24 h, and the expression of target genes was determined by WB assay. *p < 0.05, **p < 0.01.

In addition to its internalization by macrophages, FMT-β-glucan might exert its activity by binding to receptors on cells 43. C-type lectin receptors (CLRs) are pattern recognition receptors mainly expressed on immune cell surfaces to mediate the inflammatory response. Several CLRs, such as Dectin-1, Dectin-2 and mincle, can induce signaling cascades to activate the NF-κB pathway in a Syk-dependent manner 44. Thus, the expression of several CLRs was examined by qRT-PCR and WB assays. The results showed that Dectin-1 was upregulated to the greatest degree following FMT-β-glucan stimulation at the transcriptional level (Figure 6A-6D).

According to our investigation, FMT+β-glucan could polarize macrophages into the M1 type by activating the MAPK and Syk/NF-κB pathways (Figure S3). Whether FMT-β-glucan could activate MAPK and NF-κB pathways and Dectin-1 was involved in the phosphorylation of components in the MAPK and NF-κB pathway was unclear. To verify the role of Dectin-1, si-Dectin-1 was used to knock down the expression of Dectin-1 in RAW 264.7 cells. The knockdown efficacy was confirmed by downregulated Dectin-1 expression at the mRNA level (Figure 6E). As shown in Figure 6F, the expression of p-P38, p-ERK, and p-JNK in the MAPK pathway and p-Syk, p-PKC 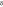, p-IKK 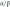, and p-P65 in the Syk/NF-κB signaling were highly elevated with FMT-β-glucan stimulation. In addition, the elevated phosphorylated proteins in the FMT-β-glucan-treated group were greater than that those in the FMT+β-glucan treatment group. Furthermore, when the Dectin-1 gene was knockdown in RAW 264.7 cells, the phosphorylation of proteins caused by FMT-β-glucan incubation was highly abrogated. Overall, Dectin-1 might play a vital role in macrophage activation induced by FMT-β-glucan.

## Discussion

Tumor cells, immune and inflammatory cells, fibroblasts, and other cells constitute a complex tumor microenvironment, in which immune cells play a vital role and significantly correlated with the progression and prognosis of patients 45, 46. Therapeutic strategies focusing on activation or modification of immune cells have been used to inhibit melanoma growth. Interleukin (IL)-2 was approved by the FDA in 1998 for metastatic melanoma therapy and can act directly on effector CD8^+^ T and regulatory CD4^+^ T cells 47. Due to adverse events such as tachycardia and multiple system organ failure and the poor response rate of patients with high serum vascular endothelial growth factor and fibronectin, IL-2 therapy has not been widely used 48. In addition, chimeric antigen receptor (CAR)-T cell therapy in which T cells are genetically engineered to recognize tumor antigens (melanoma-associated antigen-A3, mesenchymal epithelial transition, CD70, GD2, etc.) has also entered clinical trials in melanoma immunotherapy 49, 50. However, because of the self-deficiencies of CAR-T cell therapy (poor solid tumor curative effect, cytokine release syndrome, off-target effect, etc.), there was no reports of large-scale clinical treatment of melanoma by CAR-T therapy 51. Therefore, how to effectively activate the body's own immune cells that had the potential to kill tumor cells is the key to immunotherapy in melanoma.

In the tumor milieu, infiltrated macrophages initially have an M1-polarized phenotype. The classic proinflammatory M1 type exerts antigen-presenting and tumoricidal activities, partly through expressing high levels of the major histocompatibility complex class I and II molecules, and secreting cytokines (e.g., TNF-α, IL-1β and IL-6), and reactive species of oxygen and nitrogen (e.g., nitric oxide and superoxide) 52. However, continued presence in the tumor microenvironment polarizes them to M2-like tumor-associated macrophages. The M2 type macrophages lose their ability to phagocytize or present tumor associated antigens to T cells 53. Moreover, they can support cancer cell growth, silence effector immune cells, enhance angiogenesis, and promote invasion and metastasis of tumors, resulting partly from producing various mediators (tumor growth factor-β, prostaglandin E2, vascular endothelial growth factor, etc.) and enzymes (metalloproteinase 2, cathepsins, etc.) 54, 55. In melanoma, tumor-associated macrophages account for the majority of all immune cells. Therefore, polarizing tumor-associated macrophages into the antitumor M1 type could be a beneficial method to effectively treat melanoma.

Nanoparticles, generally characterized by submicronic (<1 µm) systems of metallic or organic materials, offer broad applications in various fields including tumor detection, imaging, diagnosis and treatment 56. Several types of nanoparticles, such as carbonaceous, Prussian blue, or paramagnetic nanoparticles are available for *in vitro* diagnosis based on their peroxidase-like activity or function as enzyme carriers 57, 58 and *in vivo* diagnosis as carriers of contrast agents 59, 60. In addition to diagnosis, their current applications in cancer treatment have gained widespread interest. Numerous investigations have shown that nanoparticles can control the distribution of anticancer drugs via a drug vehicle able to target tumor tissues or cells 61, 62. However, due to inappropriate pharmacokinetic properties and concerns about biodegradation, elimination and toxicity, most nanoparticles can be applied only to nanodiagnostics and not to nanotherapeutics 63. Because the release of catalytic free iron from FMT was negligible, FMT was very suitable to select as a carrier of tumor therapy agents in this study with no toxicity.

Chemotherapeutic drugs, such as doxorubicin and paclitaxel can be bound to the surface of nanoparticles and delivered to tumor sites with increased accumulation in pathological organs and protection from nonspecific toxicity 64, 65. Moreover, surface-modified nanoparticles could be rapidly opsonized and massively endocytosed/phagocytosed by cells from the mononuclear phagocyte system (bone marrow, spleen, liver, and lungs) after systemic administration, resulting in modulation of the tumor microenvironment 66. As an important participant in the tumor microenvironment, tumor-associated macrophages (TAMs) with an M2-like profile could confer to an immunosuppressive state and further promote tumor progression 67. Changing TAMs from the M2 to M1 type may provide a more effective tumor therapeutic strategy. In addition to the role of drug carriers, FMT was shown to induce macrophages into the M1 type and exerted antitumor activity in our study, which was consistent with other studies 68. After FMT stimulation, macrophages tend to develop into M1-type macrophages, accompanied by the increased expression of M1-related genes and cytokines (TNF-α and IL-6). In the presence of FMT, macrophages significantly reduced the tumor cell viability of tumor cells when directly and indirectly cocultured with tumor cells. In general, FMT can not only deliver immune activators, but can also have a synergistic effect when used in combination with other activators. β-glucan receptors including complement receptor 3 (TLR 3) and Dectin-1, are widely expressed on myeloid cells 69. By binding to these receptors, β-glucan exerts its immunomodulating effects to defend against infection and prevent tumor growth by activating various immune cells (macrophages, T cells, natural killer cells, etc.) 70, 71. Consistent with these studies, we found that solo treatment with β-glucan could indirectly inhibit B16F10 cell growth by skewing macrophages toward an M1 phenotype through activating MAPK and NF-κB signaling.

In this study, we aimed to prepare a nanocomposite of FMT-β-glucan and explore whether FMT-β-glucan had the same or better effect on immune regulation and tumor inhibition than the combination treatment of FMT plus β-glucan. Compared with FMT+β-glucan, intraperitoneal administration of FMT-β-glucan to male B16F10 bearing mice more strongly suppressed tumor growth and induced increased infiltration of M1-like macrophages in tumor tissues. *In vitro* mechanistic studies indicated that FMT-β-glucan might polarize macrophages into M1-type macrophages by binding to Dectin-1 on the surface of RAW 264.7 cells to exert a stronger toxicity to tumor cells. Collectively, this study provides a novel way to activate the immune system to maximize the efficacy of cancer therapy.

## Conclusion

In this study, a macrophage-targeted nanocomposite for melanoma therapy was synthesized. Taking advantage of the carrier function of FMT and the immune regulation of β-glucan, the nanocomposite of FMT-β-glucan displayed better anti-melanoma efficacy than the combined treatment of FMT plus β-glucan by inducing the M1 macrophage-activated tumor microenvironment. Further studies showed that FMT was engulfed by macrophages and activated the MAPK and Syk/NF-κB pathways to polarize macrophages into M1 type through the Dectin-1 receptor. M1-like macrophages induced apoptosis and cell cycle arrest of tumor cells by releasing multiple factors including TNF-α, IL-6 and ROS. Our results suggested that FMT-β-glucan might hold great promise for the clinical management of melanoma.

## Supplementary Material

Supplementary figures.

## Figures and Tables

**Table 1:** Primer sequences used in qRT-PCR

Gene name	Primer sequence (5' to 3')
**GAPDH**	
forward	AGGTCGGTGTGAACGGATTTG
reverse	TGTAGACCATGTAGTTGAGGTCA
**TNF-α**	
forward	CAGGCGGTGCCTATGTCTC
reverse	CGATCACCCCGAAGTTCAGTAG
**iNOS**	
forward	GGAGTGACGGCAAACATGACT
reverse	TCGATGCACAACTGGGTGAAC
**IL-1β**	
forward	GAAATGCCACCTTTTGACAGTG
reverse	TGGATGCTCTCATCAGGACAG
**IL-6**	
forward	AAGAAATGATGGATGCTACC
reverse	AGTTTCTGTATCTCTCTGAAG
**Arg-1**	
forward	CTCCAAGCCAAAGTCCTTAGAG
reverse	GGAGCTGTCATTAGGGACATCA
**CD206**	
forward	CTCTGTTCAGCTATTGGACGC
reverse	TGGCACTCCCAAACATAATTTGA
**p22phox**	
forward	CTACTGCTGGACGTTTCACAC
reverse	GGTGGACCCCTTTTTCCTCTT
**p40phox**	
forward	GGGCCATCATGGATCGCAT
reverse	CCAGCCAGTCTTTGTTGATCTT
**p47phox**	
forward	ACACCTTCATTCGCCATATTGC
reverse	CCTGCCACTTAACCAGGAACA
**p67phox**	
forward	GGAGAAGTACGACCTTGCTATCA
reverse	ACAGGCAAACAGCTTGAACTG
**NOX2**	
forward	AGTGCGTGTTGCTCGACAA
reverse	GCGGTGTGCAGTGCTATCAT
**Rac**	
forward	ACAGTAAGCCGGTGAACCTG
reverse	CTGACTAGCGAGAAGCAGATG
**Bcl-2**	
forward	GCTACCGTCGTGACTTCGC
reverse	CCCCACCGAACTCAAAGAAGG
**Bax**	
forward	AGACAGGGGCCTTTTTGCTAC
reverse	AATTCGCCGGAGACACTCG
**CDK2**	
forward	ATGGAGAACTTCCAAAAGGTGG
reverse	CAGTCTCAGTGTCGAGCCG
**CDK4**	
forward	ATGGCTGCCACTCGATATGAA
reverse	TGCTCCTCCATTAGGAACTCTC
**cyclin A2**	
forward	GCCTTCACCATTCATGTGGAT
reverse	TTGCTCCGGGTAAAGAGACAG
**cyclin B1**	
forward	CTTGCAGTGAGTGACGTAGAC
reverse	CCAGTTGTCGGAGATAAGCATAG
**Dectin-1**	
forward	AAAGCCAAACATCGTCTCACC
reverse	CGAGTTGGGGAAGAATGCTGAT
**Dectin-2**	
forward	TCCACAAGGTAATGGCAAATGG
reverse	CTATTGAAACACACCGCTCTTCT
**Dectin-3**	
forward	ACCCGACATCCCCAACTGAT
reverse	CTCTCGTCCAGCGTAAAAAGT
**mincle**	
forward	TGTCGTAACATATCGCAGCTC
reverse	GGACAGCAATTCTTGACTGAACC

## Abbreviations

FMT: Ferumoxytol
IL-2: Interleukin-2
CAR-T: chimeric antigen receptor-T
TNF-α: tumor necrosis factor-α
TLR 3: toll-like receptor 3
FMT surface-coated with β-glucan: FMT-β-glucan
MAPK: mitogen-activated protein kinase
Syk: spleen associated tyrosine kinase
PKC δ: protein kinase C delta
NF-κB: nuclear factor kappa-B
DMEM: Dulbecco's modified Eagle's medium
M-CSF: macrophage colony-stimulating factor
CCK-8: Cell Counting Kit-8
qRT-PCR: quantitative real time-polymerase chain reaction
ELISA: enzyme linked immunosorbent assay
PBS: phosphate buffered solution
ROS: reactive oxygen species
DCFH-DA: 2,7-Dichlorodi-hydrofluorescein diacetate
FCM: flow cytometry
WB: Western blotting
BSA: bovine serum albumin
TBST: Tris-buffered saline with 0.1% Tween 20
iNOS: inducible nitric oxide synthase
RhB: Rhodamine B
DAPI: 4,6-diamino-2-phenyl-indole
siRNA: small interfering RNA
IHC: Immunohistochemistry
NADPH: Nicotinamide adenine dinucleotide phosphate
DLS: Dynamic light scattering
TAMs: tumor-associated macrophages
M-MDSCs: myeloid-derived suppressor cells
