# Supplementary material for: Ferumoxytol-β-glucan Inhibits Melanoma Growth via Interacting with Dectin-1 to Polarize Macrophages into M1 Phenotype
Source: Int J Med Sci. 2021 Jun 26;18(14):3125–39. doi: 10.7150/ijms.61525 (PMC8364471; doi:10.7150/ijms.61525)
Supplement: Supplementary file 1 — Supplementary figures. [file ijmsv18p3125s1.pdf]

Figure S1. FMT+β-glucan regulated macrophages *in vivo*. (A-D) Quantitative data of FCM analysis of MDSCs (CD11b^{+}Gr-1^{+}; A), macrophages (CD11b^{+}F4/80^{+}; B), M1 (CD45^{+}CD11b^{+}F4/80^{+}iNOS^{+}; C) and M2 (CD45^{+}CD11b^{+}F4/80^{+}CD206^{+}; D) macrophages in bone marrow cells of mice with indicated treatments. * $P < 0.05$, ** $P < 0.01$. (E-H) Quantitative data of FCM analysis of MDSCs (CD11b^{+}Gr-1^{+}; E), macrophages (CD11b^{+}F4/80^{+}; F), M1 (CD45^{+}CD11b^{+}F4/80^{+}iNOS^{+}; G) and M2 (CD45^{+}CD11b^{+}F4/80^{+}CD206^{+}; H) macrophages in splenocytes of mice with indicated treatments.

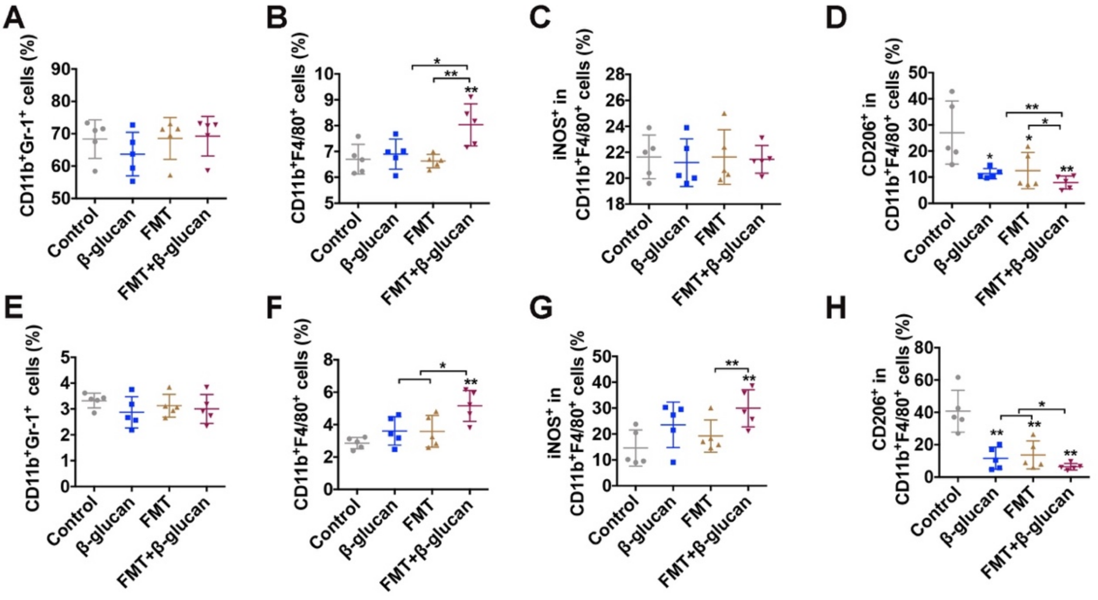

Figure S2. Effect of FMT+β-glucan on tumor growth inhibition of ID8 cells. (A) ID8 cells were treated with FMT, β-glucan, or FMT+β-glucan for 48 h and the cell viability was examined by CCK-8 assay. (B) In the presence of FMT, β-glucan, or FMT+β-glucan, ID8 cells were directly co-cultured with RAW 264.7 cells for 48 h and the proliferation of ID8 cells was detected by FCM. (D) Quantitative data of (C). (E) Schematic diagram of the indirect co-culture system. (F) In the presence of FMT, β-glucan, or FMT+β-glucan, ID8 cells were indirectly co-cultured with RAW 264.7 cells for 48 h and the cell proliferation of ID8 cells was detected by FCM. (G) Quantitative data of (F). (H) After FMT, β-glucan, or FMT+β-glucan treatment for 24 h, the cell culture supernatants of RAW 264.7 cells were collected and used to treat ID8 cells for 48 h. The cell viability of ID8 cells was detected by CCK-8 assay. * $p < 0.05$, ** $p < 0.01$.

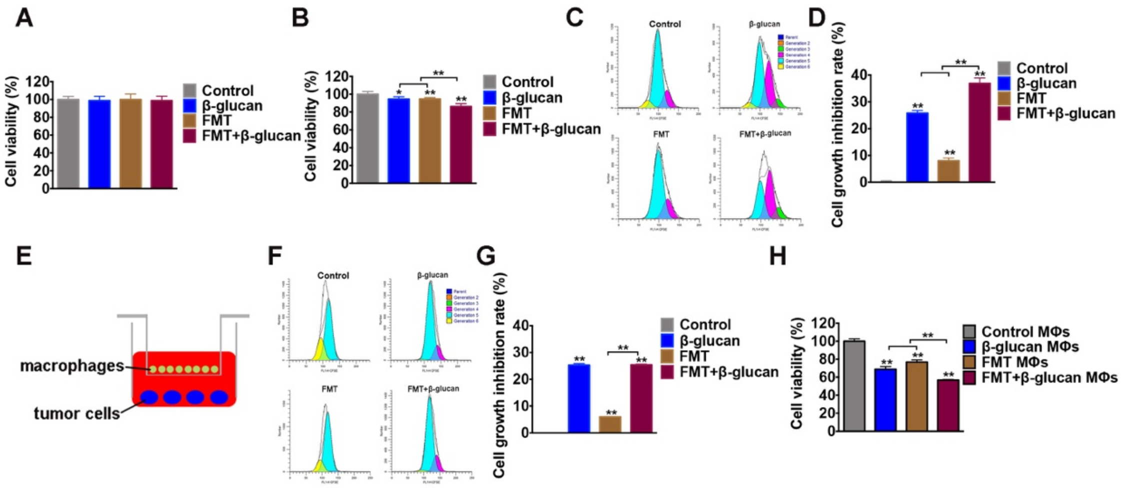

Figure S3. FMT+β-glucan polarized RAW 264.7 cells into M1 type by MAPK and Syk/NF-κB pathway. (A) RAW 264.7 cells were treated with different concentrations of FMT or β-glucan for 48 h and the cell viability was examined by CCK-8 assay. (B) After the treatment of FMT, β-glucan or their combination for 6 h, the M1-like gene expression of RAW 264.7 cells was detected by qRT-PCR experiment. (C) After the treatment of FMT, β-glucan or their combination for 6 h, the M2-like gene expression of RAW 264.7 cells was detected by qRT-PCR experiment. (D) RAW 264.7 cells were treated with FMT, β-glucan or FMT+β-glucan for 24 h and the TNF-α and IL-6 production in the cell culture supernatant was detected by ELISA experiment. (E) Phagocytic activities of RAW 264.7 cells were detected by the neutral red uptake assay after FMT, β-glucan or FMT+β-glucan incubation for 24 h. (F) RAW 264.7 cells were incubated with FMT, β-glucan or FMT+β-glucan for 12 h and intracellular ROS production was detected by FCM. (G) Quantitative data of (D). (H) RAW 264.7 cells were treated with FMT, β-glucan or FMT+β-glucan for 24 h and the expression of target proteins was analyzed by WB assay. (I) RAW 264.7 cells were incubated with FMT, β-glucan or FMT+β-glucan for 24 h and p65 translocation was visualized by confocal microscopy. (J, K) RAW264.7 cells were pre-treated with KA for 2 h and then treated with KA plus FMT, β-glucan or FMT+β-glucan for 6 h, and the expression of TNF-α (J) and IL-1β (K) was detected by qRT-PCR assay. * $p < 0.05$, ** $p < 0.01$.

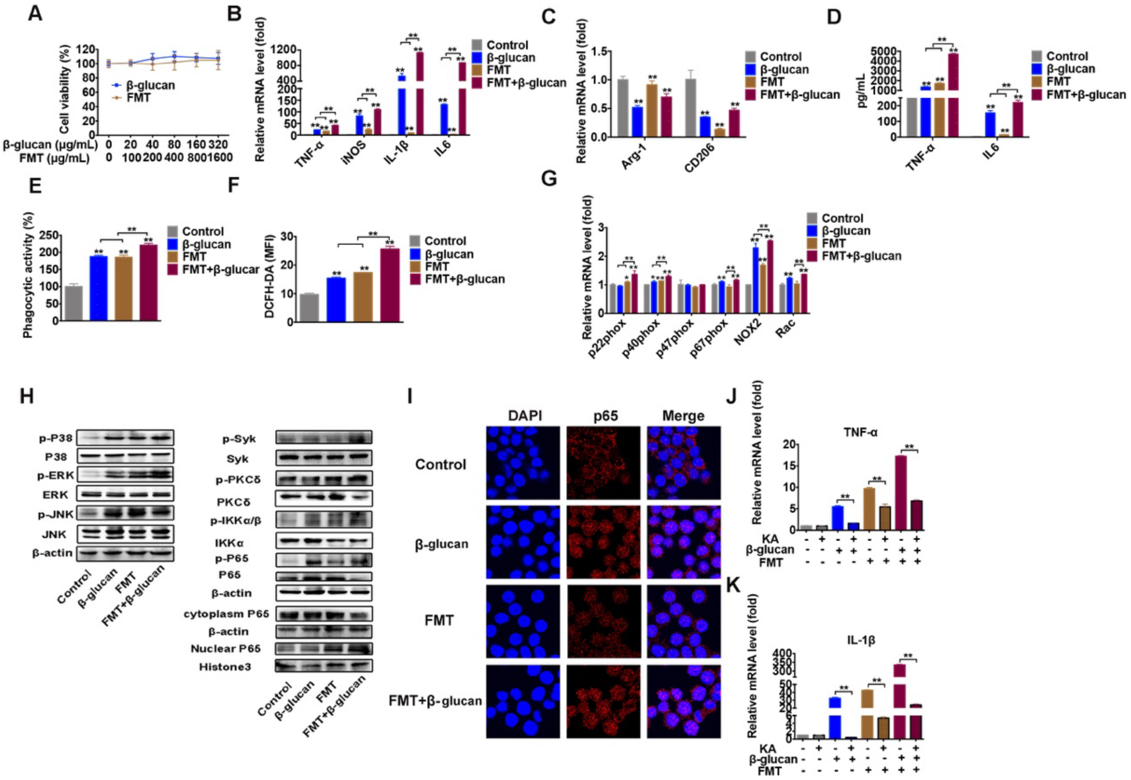

A
Cell viability (%)
β-glucan
FMT
β-glucan (μg/mL) 0 20 40 80 160 320
FMT (μg/mL) 0 100 200 400 800 1600
B
Relative mRNA level (fold)
TNF-α
iNOS
IL-1β
IL6
Control
β-glucan
FMT
FMT+β-glucan
C
Relative mRNA level (fold)
Arg-1
CD206
D
pg/mL
TNF-α
IL6
E
Phagocytic activity (%)
F
DCFH-DA (MFI)
G
Relative mRNA level (fold)
p22phox
p40phox
p47phox
p67phox
NOX2
Rac
H
p-P38
P38
p-ERK
ERK
p-JNK
JNK
β-actin
p-Syk
Syk
p-PKCδ
PKCδ
p-IKKα/β
IKKα
p-P65
P65
β-actin
cytoplasm P65
β-actin
Nuclear P65
Histone3
I
DAPI
p65
Merge
Control
β-glucan
FMT
FMT+β-glucan
J
TNF-α
Relative mRNA level (fold)
KA
β-glucan
FMT
K
IL-1β
Relative mRNA level (fold)
KA
β-glucan
FMT
